# Renin-Angiotensin System: Updated Understanding and Role in Physiological and Pathophysiological States

**DOI:** 10.7759/cureus.40725

**Published:** 2023-06-21

**Authors:** Ashok Kumar Kanugula, Jasleen Kaur, Jaskaran Batra, Anvitha R Ankireddypalli, Ravikanth Velagapudi

**Affiliations:** 1 Department of Internal Medicine, Wellstar Health System - Spalding Regional Hospital, Griffin, USA; 2 Department of Endocrinology, Diabetes, and Metabolism, HealthPartners, Minneapolis, USA; 3 Department of Internal Medicine, Univerity of Pittsburg Medical Center (UPMC) McKeesport, McKeesport, USA; 4 Department of Endocrinology, University of Minnesota School of Medicine, Minneapolis, USA; 5 Department of Pulmonary and Critical Care Medicine, Spectrum Health/Michigan State University, Grand Rapids, USA

**Keywords:** cardio-protection, cardiovascular, hypertension, obesity, renin-angiotensin-aldosterone system

## Abstract

The classical view of the renin-angiotensin system (RAS) is that of the circulating hormone pathway involved in salt and water homeostasis and blood pressure regulation. It is also involved in the pathogenesis of cardiac and renal disorders. This led to the creation of drugs blocking the actions of this classical pathway, which improved cardiac and renal outcomes. Our understanding of the RAS has significantly expanded with the discovery of new peptides involved in this complex pathway. Over the last two decades, a counter-regulatory or protective pathway has been discovered that opposes the effects of the classical pathway. Components of RAS are also implicated in the pathogenesis of obesity and its metabolic diseases. The continued discovery of newer molecules also provides novel therapeutic targets to improve disease outcomes. This article aims to provide an overview of an updated understanding of the RAS, its role in physiological and pathological processes, and potential novel therapeutic options from RAS for managing cardiorenal disorders, obesity, and related metabolic disorders.

## Introduction and background

The renin-angiotensin system (RAS) is a complex hormonal pathway that is a critical regulator of blood volume, electrolyte balance, and systemic vascular resistance. The classical understanding of RAS is that it comprises three significant components: renin, angiotensin II, and aldosterone [1, 2]. The discovery of new peptides in the last few decades has increased our understanding and the complexity of the RAS [3]. These new peptides form a counter-regulatory pathway of the RAS, which oppose the actions of the classic arm [4]. More recent studies continue to enhance our understanding of the newer peptides in the RAS and cross-talk between the two main pathways and their receptors.

The RAS is ubiquitous with the involvement of multiple organ systems, especially the kidneys, lungs, systemic vasculature, adrenal cortex, and brain [3]. The traditional view of circulating RAS is that of an endocrine system. Discoveries over the last few decades have led to a change in this view. Recent findings show that RAS components are produced in various tissues around the body, with paracrine and autocrine effects in these tissues [5, 6]. Local or tissue RAS (tRAS) is present in the heart, kidneys, adrenals, blood vessels, brain, adipose tissue, ovaries, testes, and skin [7, 8]. 

Both circulating and tRAS have a significant role in the physiological process of salt and water homeostasis, blood pressure regulation, and multiple cellular processes in different organ systems. They also have a significant role in pathophysiological conditions of hypertension, heart failure, other cardiovascular diseases, renal diseases, obesity, and metabolic disorders [5, 9]. In addition to describing the classical pathway of RAS, a novel and more detailed understanding of the counter-regulatory pathway of RAS will be discussed in this article. 

## Review

Classical pathway

Prorenin

Prorenin is an inactive precursor of renin, consisting of prosegment with 43 amino acids at the N-terminal compared to renin [10]. Many tissues in the body constitutively release prorenin. So far, no acute processes are known to induce the release of prorenin. Prorenin is produced in multiple tissues, including juxtaglomerular cells in the kidney, adrenal gland, placenta, uterus, retina, testes, and submandibular glands (also a part of local RAS) [11, 12]. The concentration of prorenin in the blood is thought to be ten times higher than that of renin under normal circumstances [13]. This concentration increases to 40-200 times that of renin in patients with diabetes complicated by retinopathy, nephropathy, and pregnancy [14-16]. The function of blood prorenin is not yet known [15].

Renin

Within the afferent arterioles of the kidney, specialized cells called juxtaglomerular (JG) cells contain prorenin. While prorenin is secreted constitutively in its inactive form, activation of JG cells causes the cleavage of prorenin to renin. This activation of prorenin to renin can be proteolytic or non-proteolytic [17]. The proteolytic activation of prorenin occurs in the kidney by enzymes like proconvertase 1 and cathepsin B [18, 19]. Mature renin is then stored in the granules of the JG cells and released into circulation by specific stimuli. 

There are four primary stimuli for the release of renin [20-22]: 1) Changes in renal perfusion perceived by the pressure transducer mechanism in afferent arterioles (sense stretch from the mechanoreceptors of the arteriolar wall); 2) Delivery of NaCl (sodium chloride) to the distal convoluted tubule that is sensed by the chemoreceptors in the macular densa cell (which forms the JG apparatus with the JG cells in the afferent arteriole); 3) Increased beta-sympathetic flow acting through the beta-1 adrenergic receptors, particularly in the upright posture; 4) Negative feedback from humoral factors like angiotensin I, potassium (renin release is increased by hypokalemia and decreased by hyperkalemia), and ANP (atrial natriuretic peptide). Therefore, conditions leading to decreased renal perfusion and reduced tubular sodium content lead to renin enzyme release into the bloodstream. The half-life of plasma renin activity is 10-15 minutes [23]. Renin is the rate-limiting enzyme in RAS [24].

(Pro)renin receptor ((P)RR)

(P)RR is an ancient molecule present in nematodes, fish, and other mammals [25]. Its structure is highly conserved, with high structural homology between species [25]. It is ubiquitously expressed as a 350 amino acid, single transmembrane protein (also a part of local RAS) [26]. It was first cloned in 2002 [27]. (P)RR has multiple ligands that induce intracellular signaling pathways [28]. Both renin and prorenin bind to (P)RR, with prorenin having a greater affinity to bind to this receptor than renin [27, 29, 30]. When renin binds to (P)RR, it undergoes a conformational change, allowing the substrate (angiotensinogen) to attach. The attachment of renin to the (P)RR also increases its catalytic efficiency by fourfold to convert angiotensinogen to angiotensin I (Ang I) [27]. Besides its role in the RAS, this receptor has been demonstrated to play a role in many physiological functions (via different ligands), including the cell cycle through cell proliferation and differentiation, acid-base balance, autophagy, energy metabolism, T-cell homeostasis, embryonic development, blood pressure regulation, water balance, and maintenance of podocyte function [25, 26, 28, 31-33]. 

Studies have demonstrated prorenin and renin to affect disease pathogenesis independent of Ang II significantly and are receptor-mediated. In the presence of an Ang II type 1 receptor blocker (losartan), these molecules activate various mitogen-activated protein (MAP) kinases (p38 and P42/44). Activating the p38 MAPK/HSP27 pathway resulted in alterations in the actin filaments in cardiomyocytes, which could explain their role in hypertrophy and cardiomyopathy [34]. In the presence of a direct renin inhibitor (remikerin), Ang II type 1 receptor blocker (losartan), and angiotensin-converting enzyme blocker (enalapril), activation of (P)RR by renin in mesangial cells was shown to induce the p42/44 MAPK pathway [35]. This led to increased production of the fibrogenic cytokine transforming growth factor (TGF)-beta, which is also known to be directly induced by Ang II via the angiotensin type 1 receptor [36-38]. Renin also led to increased levels of plasminogen activator inhibitor-type 1 (PAI-1), collagen 1 messenger RNA (mRNA) and protein, and fibronectin (FN), which are known to induce fibrosis and apoptosis. This Ang II independent effect has been seen even within the central nervous system, with activation of the (P)RR by prorenin and renin leading to increase sympathetic outflow with the possible pathogenesis of resulting hypertension [39, 40].

Angiotensinogen

This molecule is a 485 amino acid alpha 2-globulin primarily synthesized and constitutively secreted by the liver. However, angiotensinogen mRNA has been found in many other tissues (as a part of local RAS), including the heart, brain, kidney, adrenal gland, placenta, ovary, vascular and adipose tissues [41, 42]. Renin cleaves this large molecule's N-terminal and leads to the formation of angiotensin I.

Angiotensin I (Ang I)

This is considered a biologically inert decapeptide, Ang (1-10) [43].

Angiotensin-Converting Enzyme 1 (ACE1)

This enzyme is an exopeptidase expressed on plasma membranes of vascular endothelial cells, mainly in pulmonary circulation [44]. It cleaves the two amino acids from the dipeptide Ang I (1-10) carboxy-terminal to make the octapeptide Angiotensin II or Ang II (1-8). This enzyme is also expressed in intestinal and urogenital tracts, various tissues within the heart, adipose tissue, and neuronal cells in the brain [44-47].

Angiotensin II (Ang II)

Ang II is the primary mediator of the physiological effects of RAS, including volume regulation, blood pressure, and aldosterone secretion [48]. However, the half-life of Ang II in circulation is very short (<60 seconds) [49]. This raises the possibility of Ang II being produced close to the site of action, possibly functioning as part of local RAS in different tissues [9].

The physiological effects of Ang II on extracellular volume and blood pressure regulation are mediated in six ways: 1) Inducing vasoconstriction by contraction of the vascular smooth muscle [50]; 2) Activation of Na-H exchanger on the proximal tubular cells, causing an increase sodium reabsorption [51, 52]; 3) Aldosterone secretion from the adrenal cortex in the zona glomerulosa [50, 53]. This is mediated through the transcription of CYP11B2 (which encodes aldosterone synthase) [54]; 4) Increasing central sympathetic outflow and stimulatory action on the sympathetic ganglia [55]; 5) Increasing release of catecholamines from the adrenal medulla and direct ganglion stimulation [56, 57]; and 6) Increasing release of vasopressin from the hypothalamus-posterior pituitary [58].

Ang II is implicated in many pathophysiological states and is known to induce oxidative stress, vascular smooth muscle contraction, endothelial dysfunction, fibrosis, and hypertrophic, anti-apoptotic, and pro-mitogenic effects [59-61]. Ang II has been implicated in the pathogenesis of hypertension, atherosclerotic disease, heart failure, obesity-mediated hypertension, and kidney disease through these effects [5, 62-65]. In obese individuals, high angiotensin II levels lead to increased body fat accumulation and the development of insulin resistance [66]. Weight loss was associated with reduced circulating Ang II levels [66].

Ang II's physiological and pathophysiological effects are mediated by two types of Ang receptors: type 1 and type 2 [67]. These receptors have different and often opposing physiological responses attributed to the cell signaling pathways: the AT1-R receptor stimulates protein phosphorylation, and the AT2-R receptor stimulates dephosphorylation [68].

Angiotensin II Type 1 Receptor (AT1-R)

**Figure 1:**
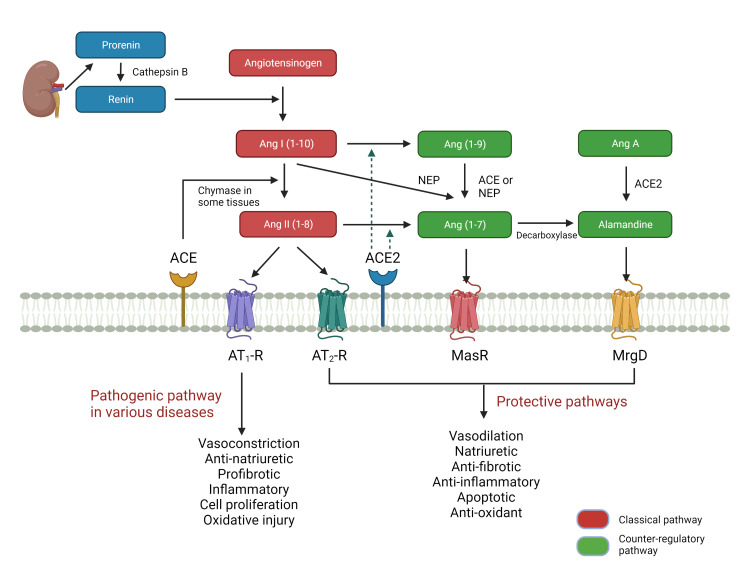
Classical and counter-regulatory pathways in the renin-angiotensin system. Ang: Angiotensin; ACE: Angiotensin-converting enzyme; NEP: Neutral endopeptidase; AT-R: Angiotensin receptor; MrgD: Mas-related G-protein coupled receptor, member D; MasR: Mas receptor. Created using BioRender.com

It is a G-protein coupled receptor [69]. It is widely distributed in many cell types, including the heart, vasculature, kidney, adrenal glands, pituitary, and central nervous system [70-73]. Ang II mediates its physiological effects of vasoconstriction and sodium and water reabsorption through the AT1-R [74]. In pathogenic states, the activation of the AT1-R leads to inflammation, fibrosis, oxidative stress, tissue remodeling, and increased blood pressure [75]. Activation of this receptor leads to activation of ADAM17, a disintegrin and metalloproteinase 17), which leads to multiple intracellular cascade effects. ADAM17 increases the transcription of EGFR (epidermal growth factor receptor), which leads to the activation of other downstream kinases, leading to increased fibrosis and vascular remodeling [74, 76]. ADAM17 produces TNF (tumor necrosis factor) alpha in the kidney, leading to an elevation in blood pressure [77]. ADAM17 cleaves and inactivates ACE2, thereby counteracting the beneficial effects of the counter-regulatory pathway, which is central to balancing the pathogenic aspects of the classical RAS pathway (Figure 1) [78]. The dysregulation of this receptor is central to the pathophysiology of cardiac and renal diseases [74, 79, 80]. This receptor is selectively blocked with angiotensin-receptor-blocking drugs or “-sartans.”

Angiotensin II Type 2 Receptor (AT2-R)

It is a G-protein coupled receptor [69]. This receptor has a lower degree of expression compared to AT1-R. It exhibits only about 34% homology to AT1-R [81]. It is widely expressed in fetal tissues, and this expression decreases significantly in adult life [68, 82]. In adults, it is expressed in the heart, kidney, adrenal glands, and brain [83-85]. Despite its low expression, it is essential in mediating protective and opposing effects to Ang II via the AT1-R. These actions inhibit inflammation, fibrosis, and central sympathetic outflow and cause vasodilation and neuroregeneration [86, 87]. Stimulation of the AT2-R by Ang II leads to vasodilation and natriuresis, opposite to the vasoconstriction and anti-natriuresis caused by Ang II via the AT1-R [68, 88, 89].

Aldosterone

This steroidogenic hormone is synthesized in the zona glomerulosa of the adrenal cortex. The synthesis and secretion of this hormone are mainly regulated by angiotensin II, ACTH (adrenocorticotropic hormone), and extracellular potassium concentration [90, 91]. The effects of aldosterone are mediated through nuclear cytosolic receptors [92]. Mineralocorticoid receptors (MR) are found in the kidney and colon epithelial cells (for sodium transport) and the non-epithelial tissues in the heart and brain [93]. The half-life of aldosterone in plasma is less than 20 minutes [94].

Epithelial effects (classic effects): Aldosterone mediates its effects on electrolyte and water homeostasis by binding to the MR receptors on principal epithelial cells in the renal cortical collecting duct. Sodium is reabsorbed via the ENaC (epithelial sodium channel) on the apical membranes of principal cells in the collecting tubules. Aldosterone inactivates Nedd 4-2 (neural-precursor-cell-expressed, developmentally downregulated gene 4-2), which degrades ENaC [95, 96]. Aldosterone increases transcription of Sgk1 (serum and glucocorticoid inducible kinase 1), leading to the inactivation of Nedd 4-2 by phosphorylation - the number of active ENaC increase, leading to increased reabsorption of sodium [94, 97]. Aldosterone activates Na-K ATPase at the apical cells' basolateral membrane [98]. This leads to sodium transport in the extracellular space and increases potassium uptake in the apical cells. 

Non-epithelial effects (non-classic effects): The effects of aldosterone on the cardiovascular and central nervous systems are complex and multifaceted. 1) Cardiovascular system: Aldosterone can increase vascular tone, potentially by increasing the production and sensitivity of catecholamines and decreasing responsiveness to the vasodilatory effects of acetylcholine [99, 100]. It also leads to the upregulation of Ang II receptors [99]. At physiologic levels, aldosterone induces the production of nitric oxide synthase [101]. Aldosterone can impair vascular function at pathogenic levels by reducing nitric oxide availability [101-103]. Aldosterone promotes the growth and remodeling of cardiac cells, which can lead to the development of cardiac inflammation, hypertrophy, and fibrosis [104, 105]; 2) Central nervous system: The effects of MR on the brain are context-dependent. We will limit the discussion to salt and water homeostasis. MRs are highly expressed in regions that regulate stress responses, including the hippocampus, amygdala, cerebellum, and prefrontal cortex [106, 107]. These regulate thirst, salt appetite, and blood pressure [108, 109].

Counter-regulatory (protective) pathway

Angiotensin-Converting Enzyme 2 (ACE2)

The discovery of this enzyme was first reported in 2000 [110]. This enzyme is a monocarboxypeptidase with the closest structural homology to ACE1 [111]. The catalytic domain of the ACE2 enzyme is 42% similar to ACE1 [110]. Two substrates of ACE2 in the RAS are Ang I (1-10) and Ang II (1-8). The catalytic efficiency of ACE2 is 300 times higher for Ang II than Ang I [111]. ACE2 is also widely expressed in the lungs, cardiovascular system, kidneys, adipose tissue, and brain [112-115]. The membrane-bound ACE2 levels are regulated by a metalloproteinase ADAM17, which cleaves it and creates a soluble ACE2 [116, 117]. Ang II (via AT1-R activation) is known to upregulate ADAM17 activity and decrease the activity of membrane-bound ACE2, potentially enhancing pathogenic disease processes [74, 118].

Severe acute respiratory syndrome coronavirus 1 (SARS-CoV-1), which was responsible for the 2002-2004 SARS epidemic, was found to use ACE2 as its functional receptor. Previous studies have shown a significant amount of surface expression of ACE2 on lung alveolar epithelial cells and enterocytes of the small intestine [114]. These findings of tissue distribution helped us understand the disease pathogenesis. During the COVID-19 (coronavirus disease 2019) pandemic that started in 2019, the SARS-CoV-2 virus was also seen to use the ACE2 protein as its functional receptor [119, 120].

Angiotensin (1-9)

ACE2 converts Ang I (1-10) to Ang (1-9) by the hydrolysis of the C-terminal leucine [110]. This protein has been found to affect the cardiovascular system through its interaction with the AT-R [121-123]. It has anti-hypertrophic effects [121, 122, 124, 125]. Furthermore, it reduces hypertension-induced cardiovascular and renal inflammation [124, 126]. In addition, it causes blood pressure reduction through different mechanisms like vasodilation and natriuresis in experimental models [124, 126].

Angiotensin (1-7)

This peptide is the most important active product of ACE2. It has been determined to be a critical regulator of ACE2-mediated counter-regulation or protective pathway of RAS [3]. It is formed by the hydrolysis of C-terminal phenylalanine from Ang II (1-8) [127, 128]. Ang (1-7) can also be produced by NEP (neutral endopeptidase) activity on Ang I (1-10) and by propyl carboxypeptidase on Ang II (1-8) [129]. The half-life for circulating Ang (1-7) is approximately 10 seconds due to rapid metabolism by peptidases such as ACE 1 and dipeptidyl peptidase 3 [130-132]. Ang (1-7) has multiple biological activities opposing Ang II (1-8) actions [133-135]. It induces anti-inflammatory, vasodilatory, antiangiogenic, antihypertensive, and antifibrotic effects by binding to its G-protein-coupled receptor, MasR [136, 137]. Ang-(1-7) also improves lipid and glucose homeostasis [138]. In addition, Ang (1-7) prevented the development of left ventricular systolic or diastolic dysfunction in male Sprague-Dawley rats after induction of myocardial infarction [139].

A proto-oncogene MAS1 encodes MasR [140]. MasR is expressed in the brain, testes, heart, kidney, and blood vessels [4]. The Mas-related G-protein coupled receptor, member D (MrgD), is the second receptor for Ang (1-7) [141]. Ang (1-7) increases intracellular cAMP (cyclic adenosine monophosphate) levels, phosphokinase A (PKA) activity, and cAMP response element-binding phosphorylation via its action on MasR and MrgD receptors [141]. In addition, Ang (1-7) also activates the phosphatidylinositol-3-kinase-Akt pathway leading to nitric oxide synthase activation in the heart [142, 143].

Ang (1-7) can bind to the AT1-R at very high concentrations and potentially antagonize the effects of Ang II on this receptor [144]. Another study showed blockade of phenylephrine-mediated vasoconstriction by the action of Ang (1-7) on AT1-R (in the presence of MasR and AT2-R antagonists); this effect was lost in the AT1-R knockout mice [145]. It had been postulated that some of the Ang (1-7) effects might be mediated via the AT2-R [136, 146]. This was challenged by a later study, which showed the lack of increase in intracellular cAMP mediated by Ang (1-7) using an AT2-R blocker [141]. Hence, the effect of Ang (1-7) on AT2-R remains unclear. This further proves that the interactions between different components of the RAS via different receptors are very complex.

Alatensins

This term was introduced by Santos et al. for peptides created by the decarboxylation of the aspartate (Asp) residues of the angiotensin molecules to alanine (Ala) [3]. The first described member in this group (in 2008) was an octapeptide Ang A, created by the decarboxylation of the aspartate on Ang II (1-8) residue to alanine [147]. The second member of this group (first described in 2013) is a heptapeptide, Alamandine (Ala1-Ang-(1-7)) [148].

Angiotensin A (Ang A)

The affinity of Ang A to the AT1-R is similar to that of Ang II, but it exhibits a higher affinity to the AT2-R. Ang A has a lower intrinsic activity at the AT1-R, translating to weaker vasoconstrictive and hypertensive effects [147]. Under normal conditions, the concentration of Ang A (Ala1-Ang-(1-8)) is about 20% that of Ang II (1-8) [149]. The ratio of Ang A/Ang II is significantly increased in end-stage renal disease [147]. Due to its more potent effects at the AT2-R, it may oppose the deleterious effects of Ang II [147]. The downstream impact of MasR remains to be elucidated.

Alamandine (Ala)

Two mechanisms generate this heptapeptide: 1) Action of ACE2 on Ang A and 2) Decarboxylation of aspartate residue to alanine on Ang (1-7)[148, 150]. Alamandine acts through MrgD [148]. MasR is also the functional receptor for Ala [151]. Ala leads to a reduction in blood pressure through vasodilation, and a reduction in oxidative stress and inflammation [148, 152]. Ala attenuated fibrosis and cardiac dysfunction due to chronic hypertension in preclinical models [153]. In rats, Ala reduced hypertension and renal damage from a high salt diet by inhibiting protein kinase C. Protein kinase C causes an increase in reactive oxygen species [154]. In Sprague-Dawley rats, infusion of Ala before induced global cardiac ischemia leads to attenuation of reperfusion injury by improving post-ischemia LV pressure, and coronary flow, decreasing apoptotic protein expression, and increasing anti-oxidative protein expression. Ala is protected against renal reperfusion injury and renal dysfunction induced by a high salt diet in rats [154, 155]. These effects were through the action of Ala on the MrgD receptor [156]. In addition to reducing cardiac and renal injury, Ala, via its receptor MrgD, also reduces pulmonary fibrosis by reducing oxidative damage and increasing autophagy [157]. Alamandine could have unique effects, different from those of Ang (1-7), via its action on the MrgD receptor [158].

MrgD receptor

MrgD is widely expressed in the central nervous system, heart, kidney, sensory neurons of dorsal root and trigeminal ganglia, gastrointestinal tract, respiratory tract, and skin [159-162]. However, the downstream effects of MrgD remain to be elucidated.

Local or tissue RAS (tRAS)

The tissue renin-angiotensin system (tRAS) refers to the local production and action of the renin-angiotensin system (RAS) components in tissues outside the circulating RAS. These tissue systems produce critical components of RAS's classical and counter-regulatory pathways. Angiotensin II is produced by ACE1 and chymase (ACE-independent) action in various tissues [163]. The activity of cathepsin D on angiotensinogen in some tissues creates angiotensin I and the action of cathepsin G on angiotensinogen directly makes angiotensin II [164]. Dysregulation of the tRAS has been implicated in the pathogenesis of several diseases, including hypertension, heart failure, diabetic nephropathy, and metabolic disorders, including obesity [5, 165, 166].

Heart

The role of tRAS in the heart is to regulate cellular processes in various tissues in response to different stimuli, including ischemia. Various components of RAS are produced within multiple tissues, including renin, angiotensin II, ACE1, ACE2, and Ang (1-7). The local production of angiotensin II is also thought to be regulated by enzymes such as chymase, which is abundant in cardiac mast cells, in addition to the activity of ACE1 [167, 168]. The older hearts in animal models produce Ang II (1-8) primarily through an ACE1-independent pathway via the action of chymase [163]. The heart expresses both main types of angiotensin receptors: AT1-R and AT2-R, effects of Ang II (1-8) through these receptors can lead to cardiac remodeling [169]. Components of the counter-regulatory RAS pathway also produce and affect various physiological and pathogenic processes [7, 170]. The circulating and tissue RAS are thought to exert combined effects on multiple organ systems.

In human cardiomyocytes, ACE2 is expressed in atria and ventricles, smooth muscle cells, fibroblasts, and endothelial cells [171]. In addition to the action of ACE2, Ang (1-7) can be produced in the heart from Ang I (1-10) by propyl endopeptidase and neural endopeptidase (NEP) [129]. Ang (1-7) exerts various cardio-protective effects locally in the cardiac tissues. These include anti-hypertensive, anti-proliferative, anti-fibrotic, anti-arrhythmogenic, and anti-inflammatory effects [172]. MasR expression in the heart to various physiological and pathophysiological stimuli was demonstrated in DOCA (deoxycorticosterone acetate)-salt rats [173]. MrgD receptor is expressed in cardiomyocytes in rats. Genetic deletion of MrgD in rats led to the development of cardiomyopathy, highlighting the importance of this receptor and potentially of its ligand, alamandine, in the regulation of normal cardiac function [174].

Adipose Tissue

In the adipose tissue tRAS, all components of RAS are expressed. Under physiological conditions, these components affect adipose tissue development and differentiation through autocrine/paracrine effects [175, 176]. Adipose tRAS has been involved in developing obesity and its metabolic complications. Overactivation of the RAS in the white adipose tissue has been associated with the development of obesity, insulin resistance, glucose intolerance, and hypertension [177, 178]. In humans, expression of angiotensinogen mRNA is upregulated in obese individuals, which might contribute to the increased levels of local and circulating angiotensin II levels [179]. Angiotensinogen mRNA expression is higher in visceral adipose tissue than the subcutaneous adipose tissue in obese humans [180-182]. Nutritional status and BMI positively correlated with the expression of angiotensinogen mRNA [180]. A high-fat diet upregulated its expression while fasting, caloric restriction, and weight loss decreased its expression [183-185]. Angiotensinogen deficiency in adipocytes of mice attenuated obesity-related hypertension [186]. In rats, angiotensinogen and Ang II (1-8) influence the development and differentiation of white adipose tissue by releasing prostacyclin from adipocytes through autocrine/paracrine effects [175, 187, 188].

Administration of recombinant human ACE2 (rhACE2) significantly attenuated weight gain in high-fat diet (HFD)-fed mice, irrespective of food intake. This was due to increased energy expenditure (increased expression of uncoupling protein-1) through differentiation of brown adipose tissue (BAT), increase in BAT mass, and browning of subcutaneous white adipose tissue (sWAT). Administration of rhACE2 also improved insulin sensitivity, measured by homeostasis model assessment [189]. Ang (1-7) infusion led to similar results in HFD-fed mice [190]. In a different study, Ang (1-7) administration in HFD-fed mice led to decreased visceral adipose tissue expansion and suppressed lipogenesis via the action of Ang (1-7) on the MasR [191]. The role of alamandine on adipose tissue remains to be elucidated. A recent study demonstrated increased white adipose tissue (WAT) and reduction in BAT in MrgD knockout mice [192]. Nitric oxide (NO) and 5’-AMP-activated protein kinase (AMPK) regulated glucose metabolism through increased expression and expression of GLUT4 in the skeletal muscles [193]. Alamandine leads to the activation of the NO/AMPK pathway in mice via the MrgD receptor and might have a role in glucose metabolism [194]. This needs to be determined by future studies.

Brain

In the brain tRAS, angiotensin II is produced locally from angiotensinogen, independently of the systemic RAS. Renin has been found in neurons and astrocytes. The local production of angiotensin II is thought to be regulated by brain-specific enzymes such as chymase and cathepsin G. The brain tRAS expresses both AT1-R and AT2-R, which mediate the effects of locally produced angiotensin II [195]. The components for the counter-regulatory RAS are also present in the brain, including ACE2, Ang (1-7), MasR, and MgrD [195]. This tRAS is critical in regulating various physiological processes within the central nervous system (CNS), including blood pressure control, water balance, neuroendocrine regulation, and stress responses. Dysregulation of the brain tissue RAS has been implicated in the development of various neurological disorders, including hypertension, stroke, Alzheimer's disease, and Parkinson's disease [196].

Discussion

With the discovery of new components of the RAS, we have been able to improve our understanding of the role of RAS in various physiological and pathophysiological processes. RAS has important functions and pathogenic roles in various cardiac, renal, metabolic, and neurological functions and disorders. Traditionally, the focus was on the blockade of components of the classical pathway of RAS. These RAS blockers improved disease outcomes and contributed to an improved understanding of the RAS itself.

Blockade of the Classical Pathway of RAS or ACE/Ang II/AT1-R Axis Blockade

Direct renin inhibitor: This has not improved renal or cardiovascular outcomes in patients with type 2 diabetes [197, 198]. The use of this agent remains uncommon in clinical practice due to the lack of benefit noted from clinical trials.

Angiotensin-converting enzyme inhibitors (ACEi) and angiotensin receptor blockers (ARB): These are used as first-line agents for the management of hypertension. These agents have improved cardiovascular (CV) outcomes, including reduced hospitalizations for heart failure and CV mortality [199-203]. These agents improve kidney outcomes like reduced microalbuminuria and slow the progression of chronic kidney disease and diabetic nephropathy [204-209]. These agents improved glycemic control in patients with type 2 diabetes by enhancing glucose uptake by skeletal muscle via the glucose transporter 4 (GLUT 4), thereby improving insulin sensitivity [210-212]. Interestingly, randomized controlled trials have shown a lower incidence of type 2 diabetes mellitus in individuals treated with ACEi and ARBs versus placebo [213-215]. These agents also exert positive effects on cardiac and renal outcomes through enhanced activity of the protective arm of the RAS [216, 217]. A randomized controlled trial to study the effects of olmesartan versus amlodipine in 80 patients with hypertension and type 2 diabetes showed a significant increase in ACE2 and Ang (1-7) levels in the olmesartan arm [218]. Inhibition of ACE1 in animal models leads to reduced food intake and body weight, and improved glucose tolerance and insulin sensitivity[219-221]. Large randomized controlled trials will be needed to prove these effects of ACEi on food intake, body weight, and glucose metabolism in humans.

Mineralocorticoid receptor antagonists (MRA): Spironolactone and eplerenone (steroidal MRAs) reduce hospitalizations and mortality in patients with heart failure with reduced ejection fraction [222, 223]. Finerenone (non-steroidal MRA) reduces heart failure-related hospitalizations and improves kidney outcomes in patients with diabetic kidney disease [224, 225];

Aldosterone synthase blocker: Baxdrostat, a selective aldosterone synthase inhibitor, has shown promising results in patients with resistant hypertension in a recent phase 2 clinical trial with dose-dependent reductions in blood pressure [226].

Current Therapeutic Endeavors Focus on Enhancing the Effects of the Counter-Regulatory (protective) Pathway of RAS

ACE2-Ang(1-7)-MasR axis and Ala-MrgD axis. Chymase inhibitors are another potential therapeutic option as this enzyme leads to ACE1-independent production of Ang II (1-10) in various tRAS. These agents might hold promise to improve cardiac and renal outcomes. They might also have potential in the treatment of obesity and metabolic disorders.

Current Therapeutic Options for the Treatment of Obesity Focus on Appetite Suppression

Enhancing energy expenditure by enhancing BAT mass and activity through the effects of the ACE2/Ang (1-7)/MasR axis might be a therapeutic strategy in the future. Pharmacological agents for this pathway include ACE2 activators, Ang (1-7) analogs, MasR agonists, alamandine analogs, and, MrgD receptor agonists. The use of these agents can further improve our understanding of RAS.

## Conclusions

Discoveries of new peptides have greatly expanded our understanding of the renin-angiotensin system. Recent findings have also improved our knowledge about the pathogenesis process behind various cardiovascular, renal, and metabolic diseases. This has galvanized interest in finding newer therapeutic targets in RAS to improve cardiovascular and renal outcomes and reduce the burden of obesity and metabolic disorders. Currently, available drugs block the classical pathway of RAS. Extensive research is being done to create analogs and agonists of the counter-regulatory pathway of RAS. With the creation of such agents, there is potential for multi-target drugs that block the classical pathway and activate the counter-regulatory or protective pathway.
